# Effects of Alcohol on the Human System

**Published:** 1871-01

**Authors:** 


Effects of Alchcol on the Human System.—Drs. Parkes and Wollowicz made experiments upon a healthy soldier, to determine the physiological effect of alcohol. The man was given, for several days, alcohol in increasing quantity; then, for six days, he drank nothing but water; then drank, for three days, brandy, after which he returned to water.

Their conclusions are essentially as follows: One and two ounces of alcohol per day seemed to increase the appetite. Four ounces lessened it, and larger quantities almost destroyed it. Hence, for this man two ounces was the limit of usefulness to appetite, a much smaller quantity, long continued, would possibly have lessened the appetite.

During the first day on which alcohol was taken, the pulse increased in frequency 4 per cent., and on the sixth day, 23 per cent., the mean of the six days being 13 per cent.

Alcohol neither lowered nor increased the animal heat as far as could be observed, although the thermometer was carefully noted in the rectum and axilla. The effect of brandy was not different in this regard from that of alcohol.

Although large doses of alcohol lessened the appetite, they did not seem to impede digestion; nor did they seem to lessen the chemical changes which end in the elimination of nitrogenous excreta, of phosphoric acid and free acidity. After alcohol had been omitted for several days, the last traces of it seemed to be eliminated, the heart showed signs of unusual feebleness, and when brandy was then administered, although the heart's beats were increased in frequency, they were very much more feeble than during the administration of the alcohol.

No effect on the nervous system could be noted by any change in amount of phosphoric acid, but there were marked subjective feelings. There were, when four ounces of alcohol were taken, marked symptoms of narcotism; there was a degree of heaviness, indisposition to exertion, and loss of alacrity, with slight headache, and torpor, and sleepiness.

It is clear that any amount of alcohol over two ounces would do harm to this man, but the experiments do not show that even a less quantity might not do injury were it continued day after day. It is obvious that alcohol is unnecessary for him, and that even one ounce in the twenty-four hours produced a slight effect on the heart, which would lead to alterations in circulation, and might lead to degeneration of tissue.

As to the metamorphosis of nitrogenous tissue, it seems improbable that alcohol has any effect; it appears to us unlikely that it can enable the body to perform more work on less food, though by quickening a failing heart, it may enable work to be done which otherwise could not be so."—*Glasgow Medical Journal, Etc.*

